# Characterizing the Flavor Profile and Metabolite Discrepancies of Scallion Braised Sea Cucumber Body Wall by Flavoromics and Widely Targeted Metabolomics

**DOI:** 10.3390/foods15081452

**Published:** 2026-04-21

**Authors:** Xinran Li, Jiahui Song, Enhui Ma, Qiang Geng, Songyi Lin

**Affiliations:** 1 State Key Laboratory of Marine Food Processing & Safety Control, National Engineering Research Center of Seafood, School of Food Science and Technology, Dalian Polytechnic University, Dalian 116034, China; lixr101@163.com (X.L.); s1924225665@163.com (J.S.); 18741144397@163.com (E.M.); geng17352079760@163.com (Q.G.); 2 Engineering Research Center of Special Dietary Food, The Education Department of Liaoning Province, Dalian 116034, China

**Keywords:** scallion-braised sea cucumber, widely targeted metabolomics, nonvolatile compounds, volatilomics, correlation analysis

## Abstract

This study provides a comprehensive characterization of volatile and nonvolatile compounds in scallion-braised sea cucumber by integrating solid-phase microextraction gas chromatography-mass spectrometry (SPME-GC-MS) and widely targeted metabolomics. A total of 43 volatile compounds and 1792 nonvolatile metabolites were identified, with amino acids and their derivatives being the most abundant. Multivariate statistical analysis identified 11 key aroma-active volatiles and 619 significantly differential metabolites. Correlation network analysis demonstrated that characteristic flavors were primarily formed through coordinated pathways involving protein degradation, lipid oxidation, and carbohydrate metabolism during high-temperature braising. Terpenoids from seasonings, lipid-derived aldehydes and furans, and Maillard reaction products jointly shaped the distinctive aroma profile. This work clarifies the molecular mechanisms of flavor formation in scallion-braised sea cucumber and provides theoretical support for improving flavor regulation, processing standardization, and product quality evaluation in commercial sea cucumber production.

## 1. Introduction

Sea cucumber, as an important branch of the phylum Echinodermata, is a high-protein, low-fat health food, rich in bioactive components such as peptides, saponins, and alkaloids [1]. It is also a high-value marine product with significant economic importance in global seafood markets, particularly in East and Southeast Asia. The international trade of processed sea cucumber products has shown sustained growth, with high market prices driven by strong consumer demand for their nutritional and functional properties [2,3]. However, despite its significant nutritional value, sea cucumber inherently possesses a rather bland flavor profile, lacking the characteristic aromatic compounds consumers typically expect. In China, sea cucumber is considered a premium aquatic product and is widely consumed in traditional dishes, among which scallion-braised sea cucumber is one of the most representative culinary preparations. The commercial value of sea cucumber products is strongly influenced by their flavor quality, which directly affects consumer preference and market competitiveness. Despite its economic significance, the biochemical basis underlying flavor formation in such complex culinary systems remains insufficiently understood. Therefore, investigating the flavor formation mechanisms in scallion-braised sea cucumber is not only of scientific interest but also of practical relevance for improving product quality and supporting the high-value seafood industry.

To compensate for its bland taste, seasoning-assisted cooking is commonly used to enhance the flavor of sea cucumber. During thermal processing, its body wall, dominated by collagen fibers and a porous gel-like structure, exhibits strong water absorption and adsorption abilities, enabling the incorporation of exogenous aromatic compounds from seasonings [4]. Consequently, despite the inherently mild taste of sea cucumber, its unique structure allows it to absorb the flavors of surrounding ingredients and seasonings like a sponge, thereby enhancing the overall flavor and creating a rich sensory experience. Previous studies have demonstrated that sea cucumbers can effectively absorb aromatic compounds from single-ingredient seasoning systems, such as solutions of Sichuan pepper, star anise, or scallions [5]. However, although existing studies have confirmed that sea cucumbers can effectively adsorb exogenous flavor compounds in a single seasoning, in actual cooking systems, their unique flavor usually depends on the combination of multiple seasonings. In actual cooking scenarios, multi-omics research further indicates that exogenous flavor substances and endogenous precursors (such as amino acids and lipids) jointly contribute to the formation of the characteristic aroma in traditional sea cucumber dishes. Therefore, it is necessary to conduct systematic research by combining volatileomics and widely targeted metabolomics to comprehensively elucidate the molecular basis of flavor formation in such a complex food system [6]. These interactions lead to complex physicochemical reactions such as Maillard reactions, lipid oxidation, amino acid degradation, and thermal transformations of Spice derivatives, resulting in diverse volatile and nonvolatile metabolites. The roles and diffusion behaviors of flavor compounds under such multi-component, real-world cooking systems remain poorly understood.

Scallion-braised sea cucumber represents a complex thermal processing system involving the interaction of protein-rich marine tissue with multi-component seasonings under high-temperature conditions. Unlike simple heating models, this cooking method incorporates oil-mediated heat transfer, prolonged stewing, and the presence of Allium-derived ingredients, which are rich in sulfur-containing compounds and aroma precursors [6]. The body wall of the sea cucumber is characterized by a collagen-rich, highly porous protein matrix, which provides a unique structural environment for the adsorption, retention, and transformation of exogenous flavor compounds. This makes it a suitable model for studying the interaction between protein matrices and exogenous aroma-active compounds during thermal processing [7]. Furthermore, scallion-braising involves simultaneous lipid oxidation, Maillard-type reactions, and seasoning-derived transformations, making it representative of real-world culinary systems where multiple pathways contribute to flavor formation. Therefore, this system provides a relevant and practical model for investigating the integration of endogenous and exogenous factors in flavor development in complex food matrices.

In recent years, multi-omics technologies have emerged as powerful tools for elucidating the flavor formation mechanisms in meat, fish, tea, and fermented foods. Volatilesomics comprehensively analyzes aroma-active volatile compounds, while widely targeted metabolomics enables large-scale detection of primary and secondary metabolites associated with flavor precursors [8,9,10,11,12]. Previous studies on sea cucumber flavor have primarily focused on sensory evaluation and conventional analytical techniques such as GC-MS for volatile compound identification. While these approaches have provided valuable information on key aroma compounds, they are generally limited in scope and lack the ability to comprehensively capture the complex interactions between volatile and nonvolatile metabolites. In particular, conventional GC-MS analysis mainly targets volatile compounds and provides limited insight into the underlying metabolic networks and precursor–product relationships involved in flavor formation. Similarly, sensory analysis, although important, does not provide mechanistic or compositional resolution at the molecular level. Therefore, there remains a significant gap in understanding the integrated biochemical basis of flavor formation in complex food systems such as scallion-braised sea cucumber, where multiple thermal reactions and ingredient interactions occur simultaneously. To address this gap, the present study integrates flavoromics with widely targeted metabolomics to systematically profile both volatile and nonvolatile components and to explore their potential associations. This combined approach enables a more comprehensive characterization of flavor-related compounds and provides deeper insight into the complex interactions underlying flavor development in thermally processed foods.

Therefore, this study aims to: (1) Comprehensively characterize volatile and non-volatile metabolites in commercially available scallion-braised sea cucumber using HS-SPME-GC-MS and widely targeted metabolomics; (2) Identify key aroma-active compounds (OAV > 1) and differential metabolites responsible for flavor variations among samples; (3) Elucidate metabolic pathways governing flavor formation in the muscle layer within complex seasoning systems; (4) establish correlations between aroma-active compounds and underlying metabolic processes, providing molecular insights for flavor regulation. This study pioneers an integrated volatiles-metabolomics framework to decipher flavor formation mechanisms in scallion-braised sea cucumber within a real-world multicomponent cooking environment. The findings provide theoretical foundations for enhancing flavor quality and standardizing sea cucumber processing techniques.

## 2. Materials and Methods

### 2.1. Materials and Chemicals

This study selected three local Dalian restaurants offering scallion-braised sea cucumber as experimental subjects based on market recognition and customer preference, randomly designated as SC-X, SC-Y, and SC-Z, respectively (Dalian, Liaoning, China). All sea cucumbers used were Liaoning Sea cucumbers from the Bohai Bay region, supplied by Dalian Hongfuda Trading Co., Ltd. (Dalian, Liaoning, China). Sampling was conducted on the same day, and after collection, the samples were transported to the laboratory using cold chain logistics. All raw materials used in this study were food-grade and obtained from local market in Dalian, China. These included green onions (scallion white and leaves), ginger, dried chili, peanut oil, granulated sugar, rock sugar, cooking wine, light soy sauce, dark soy sauce, oyster sauce, fish sauce, Sichuan pepper, and table salt. Additional ingredients used for broth preparation included old hens, pork knuckles, pork bones, and Jinhua ham. Starch was used for sauce thickening. All samples were transported under controlled cold chain conditions to minimize potential biochemical and microbial changes prior to analysis. Specifically, samples were packed in insulated containers with ice packs and maintained at a temperature of 0–4 °C during transportation. The middle section of each sea cucumber was dissected into three layers—skin (s), muscle (m), and tendon (t)—using a surgical knife, yielding a total of 9 experimental samples (Figure S1). The statistical analyses were conducted based on biological replicates (n = 3). All samples were stored at −80 °C until instrumental analysis. High-purity cyclohexanone (99.5%) and n-alkanes (C7–C30, 99.5%) were purchased from Sigma-Aldrich (St. Louis, MO, USA).

### 2.2. Sample Preparation

#### 2.2.1. Preparation Process of SCX Group

A mixture of scallion white and scallion leaves was selected in a ratio of 1:2. 200 g of peanut oil was heated to 180 °C, then the scallion segments and 10 g of dried chili segments were added. The heat was kept at high and the oil temperature was maintained between 170 °C and 190 °C, stir-fried for 90 s until the surface of the scallion segments quickly became charred. 30 g of granulated sugar was directly added into the pot and stir-fried for 10 s while the oil was still at high temperature until the sugar melted halfway. Then 30 mL of 52-degree or higher strong liquor was poured in. 25 mL of dark soy sauce, 8 g of salt, and 300 mL of boiling water were added. The mixture was brought to a boil over high heat, and the SCs that had been blanched in 80 °C hot water for 30 s to remove the fishy smell were added. Stir-frying continued over high heat for 2 min without covering the pot. Just before serving, the pre-prepared compound scallion oil was poured in; this compound scallion oil had been obtained by heating 30 g of peanut oil, 50 g of chopped scallion leaves, and 5 g of ginger powder at 120 °C for 5 min and then filtering. Stir-frying was carried out quickly for 10 s, and the pot was removed from the heat. 25 g of water starch (starch and water mixed in a ratio of 1:1.2) was immediately added, and the pot was tossed to thicken the sauce.

#### 2.2.2. Preparation Process of SCY Group

The cleaned SCs were placed in a clay pot, and enough pure water was added to cover them. They were slowly simmered at 85 to 90 °C for 90 min. To prepare the base broth, an old hen, pig’s feet, and Jinhua ham were taken in a ratio of 3:2:1, and cold-water equivalent to 20 times their total weight was added. The mixture was boiled at 100 °C, then the surface foam was removed, and the heat was switched to gentle with a core temperature of about 92 °C, keeping the surface of the broth gently bubbling. It was simmered for 4 h and then strained to obtain a clear broth with high gelatin content. In another pot, 100 g of peanut oil was heated to 100 °C and kept at a constant temperature for 15 min. 200 g of scallion white segments and 30 g of ginger slices were added. A clay pot was taken, half of the fried scallion and ginger were lined at the bottom, then the pre-cooked soft SCs were added, the high broth was poured in until the ingredients were fully submerged, 15 g of rock sugar was added, the pot was covered, and it was simmered at a constant temperature of 95 °C for 25 min. In another pot, the remaining 30 g of compound scallion oil was heated to 100 °C, 10 g of granulated sugar was added, and it was slowly simmered over low heat for 3–4 min until it turned amber (approximately 160 °C). Then, 20 g of cooking wine and 20 g of light soy sauce were added, and the cooked SCs and the original broth were poured in. The mixture was heated to boiling (approximately 98–100 °C) and maintained at this temperature for 3 min, during which the sauce was continuously poured over the surface of the SCs. Finally, the sauce was thickened with water starch made by mixing starch and water in a ratio of 1:1.5, in two batches. Just before serving, 5 g of freshly pressed scallion oil was added.

#### 2.2.3. Preparation Process of SCZ Group

The SCs were placed in a steamer and steamed with 100 °C saturated steam for 15 min. The compound base oil was prepared using a “three-stage” oil temperature control method. First, 200 g of peanut oil, 150 g of scallion segments, 50 g of ginger slices, and 10 g of Sichuan pepper were heated in a pot at a constant temperature of 110 °C for 8 min. Then, some of the spices were filtered out, and the oil temperature was increased to 150 °C. 100 g of fresh scallion white segments was added and fried until golden and crispy, then removed and set aside. Finally, 50 g of the compound oil was heated to 165 °C, 20 g of rock sugar was added, and it was quickly stir-fried until it turned dark red. 500 mL of high broth made by boiling old hens and pork bones for 6 h was immediately poured in, then 30 g of oyster sauce and 10 g of fish sauce were added, and the mixture was simmered for 5 min to make the compound sauce. The steamed SCs were quickly immersed in the hot compound oil at 165 °C for 10 s to form a slightly charred layer on the surface, then removed and placed in the sauce. The previously fried golden-brown scallion sections were added, and they were simmered over a low heat of 95 °C for 8 min. Finally, the sauce was thickened with a slurry made from 30 g of starch and water in a 1:1.8 ratio, and 10 g of scallion and ginger oil was drizzled over it.

### 2.3. Analysis of Volatiles in Sea Cucumber Samples Based on Headspace Solid-Phase Microextraction Coupled with Gas Chromatography–Mass Spectrometry (HS-SPME-GC-MS)

#### 2.3.1. HS-SPME

The volatiles in sea cucumber samples were analyzed using a slightly modified version of the method previously developed by our team [13]. Prior to instrumental analysis, 2.0 g of sea cucumber samples were accurately weighed and transferred into 20 mL headspace vials. An internal standard solution of cyclohexanone (20 μL, 50 mg/L) was added to each vial. The vials were then sealed with polytetrafluoroethylene (PTFE)-lined magnetic caps. An automated injection program was employed to transfer the vials to a GC incubator maintained at 60 °C for a 30 min incubation period. After the incubation period, a SPME fiber composed of DVB/CAR/PDMS (50/30 μm, Supelco, Bellefonte, PA, USA) was placed into each vial to collect the volatile compounds from the headspace for a duration of 45 min. The DVB/CAR/PDMS fiber was selected based on preliminary experiments, which demonstrated its superior extraction efficiency for a broad range of volatile compounds in seasoned sea cucumber [14]. This fiber coating combines divinylbenzene (DVB), carboxen (CAR), and polydimethylsiloxane (PDMS), providing a wide polarity range and high adsorption capacity. Therefore, it is widely used in complex food matrices and was considered suitable for comprehensive volatile profiling in this study. Following the extraction process, the SPME fibers were subsequently transferred into the GC injection port, maintained at a temperature of 240 °C, for a thermal desorption lasting 5 min.

#### 2.3.2. GC-MS Analysis

The volatiles in the samples were analyzed using an Agilent 7890B-5977B GC-MS (Agilent Technologies Inc., Santa Clara, CA, USA) equipped with a 30 m × 250 μm × 0.25 μm DB-WAX column. Helium was used as the carrier gas at 1.0 mL/min in splitless mode. The temperature program and EI conditions were carried out according to the method of Li et al. [13].

#### 2.3.3. Identification of Volatiles in Sea Cucumber Samples

Based on the approach described by Li et al. [14] the volatile compounds present in the samples were identified and measured. For qualitative analysis, the external standard method was employed using n-alkanes (C7–C30) as reference compounds. The retention indices (RIs) of the volatiles were calculated and matched against those documented in the NIST11 database (National Institute of Standards and Technology, Gaithersburg, MD, USA) for identification. For quantitative analysis, the method of internal standards was employed. The peak areas of the volatiles were compared with those of the internal standard, based on its known concentration, to achieve relative quantification.

#### 2.3.4. Calculation of Odor Activity Values (OAV) in Sea Cucumber Samples

The OAV values of the volatiles were calculated using the method described by Wang et al. [15] Volatiles with an OAV > 1 are typically considered to play a key role in shaping the characteristic flavor of the sample. The specific calculation formula is as follows:

(1) $$\mathrm{OAV}=\frac{C_{i}}{{\mathrm{OT}}_{i}}$$

The formula employs C_i_ to denote the concentration (μg/g) of the volatiles and OT_i_ to represent the odor threshold value.

### 2.4. Analysis of Metabolites in Sea Cucumber Samples

#### 2.4.1. Sample Extraction Process

Samples were pre-stored at −80 °C and then thawed in an ice bath. All subsequent experimental operations were carried out in an ice bath. The samples were ground thoroughly in liquid nitrogen, and precisely weighed (20 ± 1) mg of homogenate was transferred into the corresponding labeled centrifuge tubes. Subsequently, 400 μL of a 70% methanol-diluted dimethyl sulfoxide solution was added to each centrifuge tube. This solution was prepared as a multi-concentration gradient internal standard solution before mass spectrometry analysis. The mixture was vortexed at 2500 rpm for 5 min using a vortex mixer (Vortex-Genie G560E, Scientific Industries, Bohemia, NY, USA), and then left to stand in an ice bath for 15 min. After the extraction step, the samples were centrifuged at 4 °C and 12,000 rpm for 10 min using a refrigerated centrifuge (CR22N, Hitachi, Tokyo, Japan). Subsequently, 300 μL of the supernatant was gently transferred into a pre-labeled new centrifuge tube and incubated at −20 °C for 30 min. After the incubation process, the samples were subjected to a second centrifugation under the same experimental conditions for 3 min. Finally, 200 μL of the supernatant was transferred to a dedicated injection vial to meet the requirements for subsequent instrumental analysis.

#### 2.4.2. Ultra-High Performance Liquid Chromatography Separation

The ultra-high performance liquid chromatography (UHPLC) separation process was accomplished using the ExionLCAD UHPLC system (Sciex, Framingham, MA, USA) coupled with a QTRAP^®^ 6500+ mass spectrometer. A Waters reverse-phase HSST3C18 column (1.8 μm particle size, 2.1 × 100 mm column dimensions) was selected as the chromatographic column, and the column temperature was maintained at 40 °C to optimize the separation of compounds. The flow rate of the mobile phase was set at 0.4 mL/min. The binary mobile phase system consisted of 0.1% formic acid in water (Phase A) and 0.1% formic acid in acetonitrile (Phase B). The chromatographic program parameters were set as follows: from 0 to 0.1 min, 95% Phase A was maintained; then a linear gradient elution was implemented, and the proportion of Phase A was adjusted to 10% at 11 min; from 11 to 12 min, isocratic elution at 10% Phase A was maintained; finally, from 12 to 14 min, the initial conditions of 95% Phase A were restored through re-equilibration.

#### 2.4.3. Bipolar Ionization Mass Spectrometry

The TurboV™ ion source was used to perform electrospray ionization tandem mass spectrometry (ESI-QTRAP-MS/MS) in bipolar mode (SCIEX, Framingham, MA, USA). The core ionization parameters were set as follows: ion source temperature 500 °C, spray voltage ± 5500 V (positive/negative ion mode), gas source 1 (GSI) 55 psi, gas source 2 (GSII) 60 psi, curtain gas 25 psi, and collision gas (CAD) intensity was adjusted to the “high” setting. During system calibration, polypropylene glycol solution was used (10 mmol in QQQ mode and 100 mmol in LIT mode). Dynamic multiple reaction monitoring (dMRM) switching strategies were optimized for metabolites in specific elution periods.

#### 2.4.4. Qualitative and Quantitative Analysis of Metabolites

In the qualitative and quantitative analysis of metabolites, the acquisition and preliminary processing of raw spectral data were accomplished using Analyst^®^ 1.6.3 software (developed by Sciex). A triple quadrupole mass spectrometer was employed to detect characteristic precursor-product ion pairs, and the signal intensities were recorded in the form of centroids. MultiQuant™ 3.0.3 software was utilized to align chromatographic peaks and normalize their intensities, while a spline interpolation algorithm was applied for baseline correction. Normalized peak areas were used to calculate the relative metabolite contents.

#### 2.4.5. Sample Quality Control Analysis

##### Evaluation of Total Ion Chromatograms

To ensure the repeatability of the sample processing and detection procedures, quality control (QC) samples were prepared by mixing all the extracts. During the instrumental analysis, a QC sample was inserted after every 10 analytical samples to monitor the system’s operational status. The technical reproducibility of the metabolite extraction and detection process was determined by evaluating the overlap of total ion chromatograms of different QC samples.

##### Correlation Evaluation of Quality Control Samples

Pearson correlation coefficient analysis was conducted on the quality control samples. The closer the absolute value of the correlation coefficient |r| between the quality control samples is to 1, the higher the stability of the entire analysis process and the more reliable the obtained data [16].

##### Internal Standard Response Stability

To assess the stability of the detection process, an internal standard of known concentration can be added to the quality control samples. Stability can be evaluated by analyzing the coefficient of variation (CV) of the internal standard response values: when the CV of the internal standard response does not exceed 15%, the detection process can be considered stable, and the obtained data quality is reliable.

##### Quality Control Evaluation Based on CV Value

The CV to mean ratio is used to quantify the degree of data dispersion. In this study, the empirical cumulative distribution function (ECDF) was employed to assess the distribution pattern of CV values for metabolites within the quality control samples. The higher the proportion of metabolites with a lower CV, the better the stability of the experimental data. Specifically, when the proportion of metabolites with a CV less than 0.5 exceeds 85%, the data stability is considered good; if the proportion of metabolites with a CV less than 0.3 reaches or exceeds 75%, the data stability is at an extremely high level.

### 2.5. Statistical Analysis

In this study, the experimental unit was defined as an independent commercial product obtained from different restaurants. A total of three restaurants were selected, and samples from each restaurant were treated as biological replicates (n = 3 per group). Within each biological replicate, the sea cucumber samples were further divided into three anatomical layers, namely the outer skin layer (s), middle muscle layer (m), and inner tendon layer (t). These layers were considered as nested subsamples within each biological replicate rather than independent experimental units. For each subsample, the extraction and instrumental analysis were performed in triplicate to ensure analytical reproducibility, which were treated as technical replicates and were not used as independent observations in statistical analyses. The performance of the models was evaluated using R^2^Y (goodness of fit) and Q^2^ (predictive ability). The statistical significance of the models was further assessed using cross-validated analysis of variance (CV-ANOVA), with *p* < 0.05 considered significant.

The analysis results were generated using GraphPad Prism (GraphPad Software, San Diego, CA, USA, version 9.5.0), Chiplot (https://www.chiplot.online/#Pie-plot), and ChemDraw3D 22.0.0 (64-bit). Multivariate statistical analyses were performed using SIMCA-P software (Umetrics, version 13.0, Malmö, Sweden).

## 3. Results and Discussion

### 3.1. Analysis of the Changing Rule of Volatiles in Different SCs

#### 3.1.1. Volatile Profile Characterization of Sea Cucumber Samples

**Figure 1:**
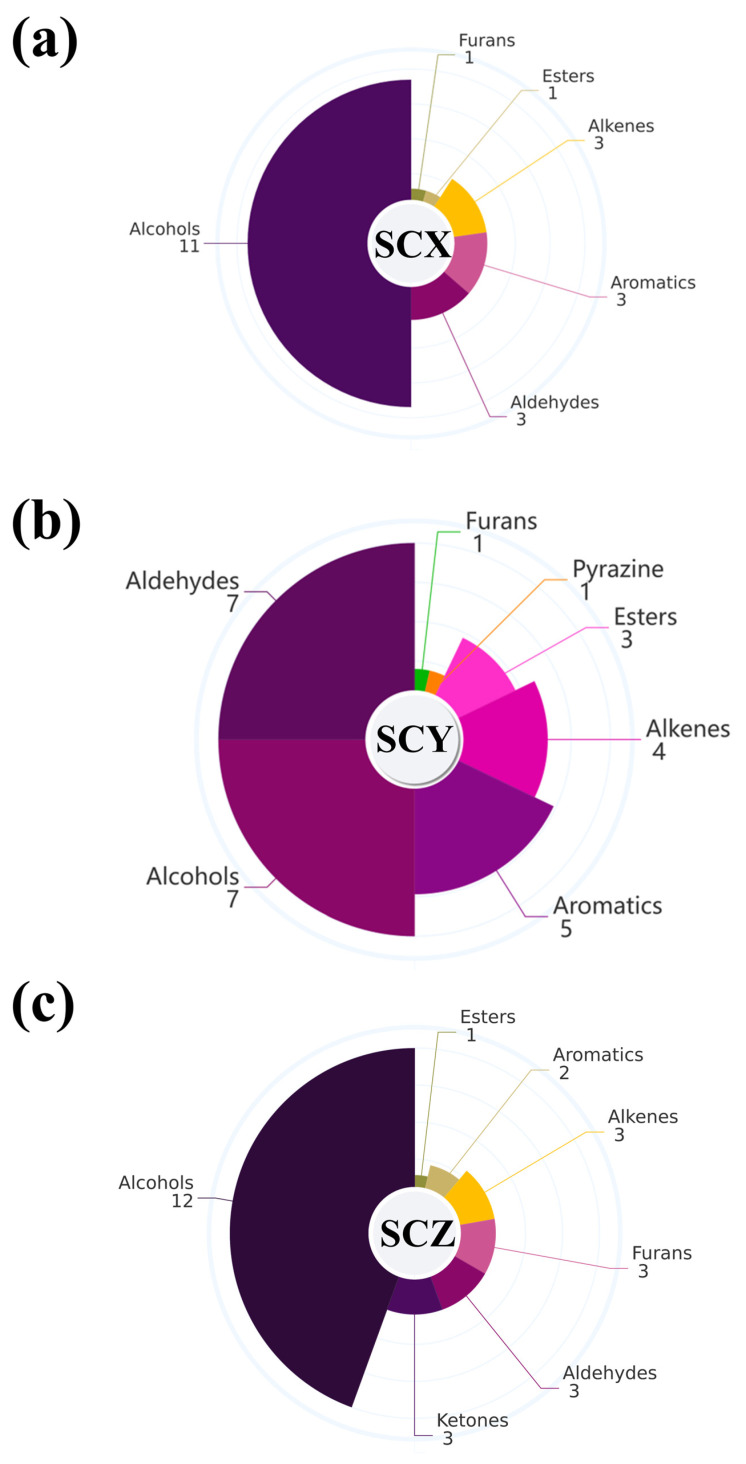

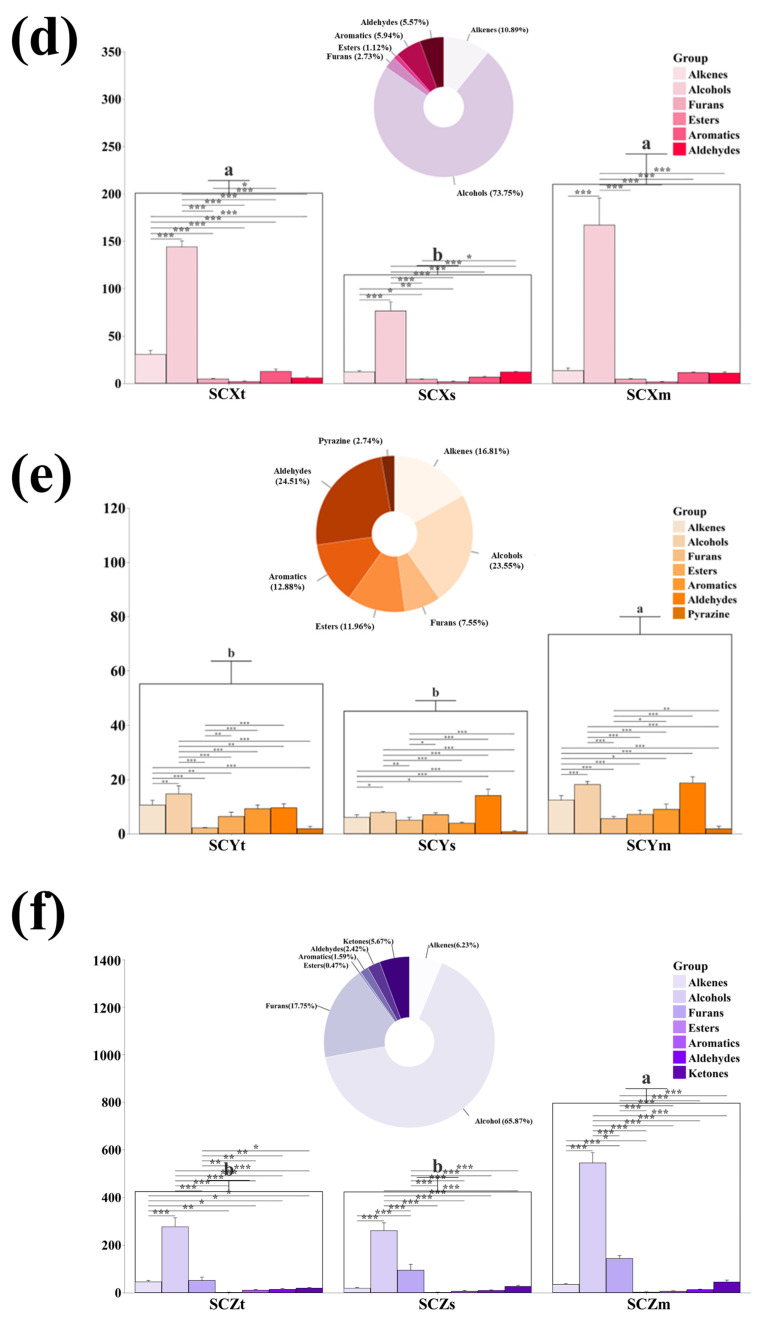

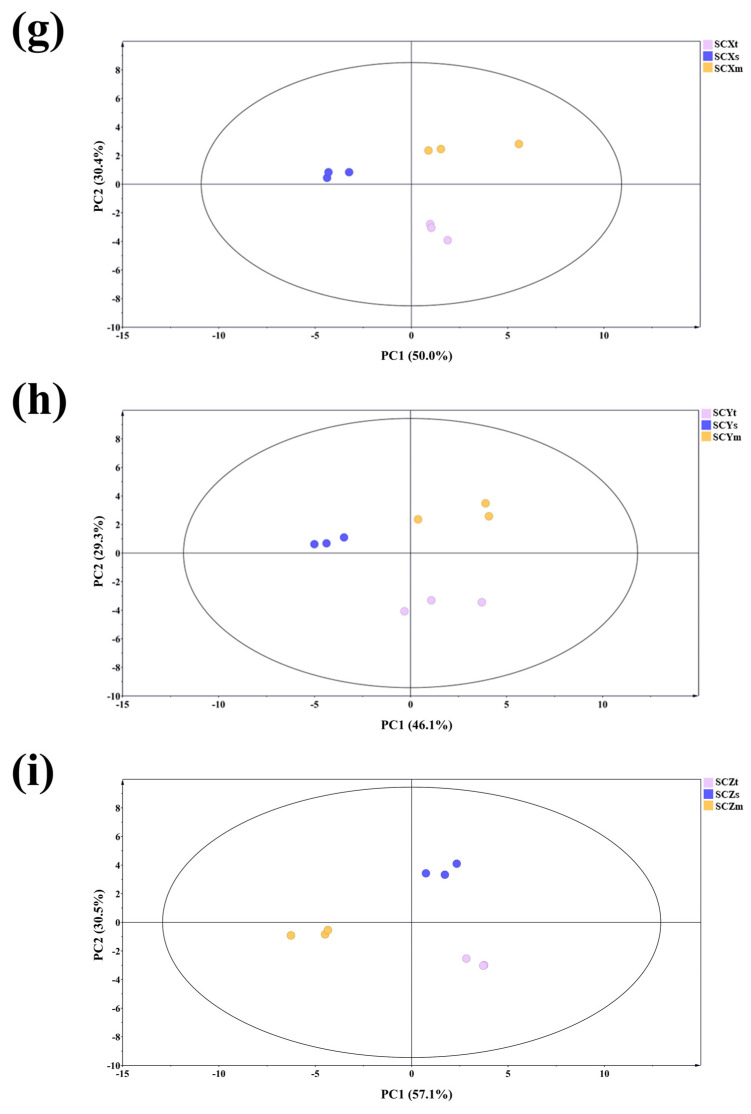
GC-MS analysis of scallion−braised sea cucumber. (**a**–**c**) Types and contents of volatile compounds. (**d**–**f**) Types and distribution of volatile compounds in different parts of scallion-braised sea cucumber. *, ** and *** indicates significant differences (*p* < 0.05) among various types of volatiles in samples. a, b indicates significant differences (*p* < 0.05) in the volatiles among different parts within the samples. (**g**–**i**) Score plots of principal component analysis of volatile compounds in different parts.

Table 1 illustrates that a total of 43 volatile compounds were found in all samples of SCs. This group consisted of 15 alcohols, 8 aldehydes, 6 aromatic substances, 4 alkenes, 3 furans, 3 ketones, 3 esters, and 1 pyrazine. While the quantity of volatiles identified in each sample group was comparable, notable variations were observed among the different groups. Specifically, SCX, SCY, and SCZ sample groups contained 22, 28, and 27 volatiles, respectively. Alcohols were the dominant chemical class in all samples, particularly in SCX (11 types) and SCZ (12 types). In contrast, SCY had 7 alcohols and an equal number of aldehydes. Additionally, no other type of volatile was detected in more than 5 types in any of the sea cucumber samples (Figure 1a–c).

These findings basically align with Zhang et al. [17] who reported that alcohols are the primary volatiles in SCs, along with aldehydes, furans, and aromatic compounds. However, the additional detection of alkenes, ketones, esters, and pyrazines in the present study suggests that scallion-braising may introduce a broader range of volatile compounds compared to raw or simply heated sea cucumber. This broader volatile profile may be associated with multiple thermal reactions, including lipid oxidation and Maillard reactions involving amino acids, peptides, and membrane lipids [18]. Allium-derived seasonings such as scallions undergo oxidation, caramelization, amino acid degradation, and additional Maillard-derived transformations during heating in the presence of oil and sugars, generating aldehydes, furans, terpenoids, and other aroma-active components that migrate into sea cucumber tissues [19]. Therefore, the more complex volatile profile observed in scallion-braised sea cucumber reflects the combined effects of endogenous thermal reactions and exogenous flavor absorption under a multi-seasoning cooking system.

**Table 1:** Volatile compounds identified in sea cucumber samples.

No.	Name	CAS	RI ^1^	RI ^2^	Molecular Formula	MW ^3^	Odor Type ^4^	Identification Methods
	**Alkenes**							
1	limonene	138-86-3	1200	1116	C10H16	136.23	citrus	MS, RI
2	styrene	100-42-5	1261	1255	C8H8	104.15	balsamic	MS, RI
3	1-tetradecen-3-yne	74752-91-3	/	1283	C14H24	192.34		MS, RI
4	caryophyllene	87-44-5	1595	1590	C15H24	204.35	spicy	MS, RI
	**Alcohols**							
5	1-hexanol	111-27-3	1355	1349	C6H14O	102.17	herbal	MS, RI
6	1-octen-3-ol	3391-86-4	1450	1450	C8H16O	128.21	earthy	MS, RI
7	2-ethyl-1-hexanol	104-76-7	1491	1487	C8H18O	130.23	citrus	MS, RI
8	2,6-dimethyl-1-nonen-3-yn-5-ol	19780-98-4	/	1527	C11H18O	166.26		MS, RI
9	2-nonanol	628-99-9	1521	1542	C9H20O	144.25	waxy	MS, RI
10	1-octanol	111-87-5	1557	1555	C8H18O	130.23	waxy	MS, RI
11	1-nonanol	143-08-8	1660	1654	C9H20O	144.25	floral	MS, RI
12	3,7-dimethyl-1-octanol	106-21-8	1666	1626	C10H22O	158.28	floral	MS, RI
13	(E)-3-decen-1-ol	10339-60-3	1765	1793	C10H20O	156.27		MS, RI
14	(Z)-3-decen-1-ol	10340-22-4	1789	1797	C10H20O	156.27		MS, RI
15	(Z)-4-decen-1-ol	57074-37-0	1791	1800	C10H20O	156.27	waxy	MS, RI
16	(E)-5-decen-1-ol	56578-18-8	1803	1805	C10H20O	156.27	waxy	MS, RI
17	(Z)-5-decen-1-ol	51652-47-2	1810	1811	C10H20O	156.27		MS, RI
18	9-decen-1-ol	13019-22-2	1813	1816	C10H20O	156.27	floral	MS, RI
19	phenylethyl alcohol	60-1-8	1906	1900	C8H10O	122.16	floral	MS, RI
	**Aldehydes**							
20	heptanal	111-71-7	1184	1180	C7H14O	114.19	green	MS, RI
21	(E)-2-heptenal	18829-55-5	1323	1322	C7H12O	112.17	green	MS, RI
22	nonanal	124-19-6	1391	1392	C9H18O	142.24	aldehydic	MS, RI
23	(E)-2-octenal	2548-87-0	1429	1425	C8H14O	126.2	fatty	MS, RI
24	furfural	98-01-1	1461	1461	C5H4O2	96.08	bready	MS, RI
25	benzaldehyde	100-52-7	1520	1514	C7H6O	106.12	fruity	MS, RI
26	benzeneacetaldehyde	122-78-1	1640	1631	C8H8O	120.15	green	MS, RI
27	(E)-2-dodecenal	20407-84-5	1867	1860	C12H22O	182.3	herbal	MS, RI
	**Furan**							
28	2-pentyl-furan	3777-69-3	1231	1225	C9H14O	138.21	fruity	MS, RI
29	trans-2-(2-Pentenyl)furan	70424-14-5	1282	1298	C9H12O	136.19		MS, RI
30	2-hexyl-furan	3777-70-6	1321	1330	C10H16O	152.23		MS, RI
	**Ketone**							
31	2-nonanone	821-55-6	1390	1387	C9H18O	142.24	fruity	MS, RI
32	2-undecanone	112-12-9	1598	1596	C11H22O	170.29	fruity	MS, RI
33	6-methyl-3-(1-methylethyl)-7-oxabicyclo [4.1.0]heptan-2-one	5286-38-4	1711	1719	C10H16O2	168.23		MS, RI
	**Ester**							
34	2,2,4-trimethyl-1,3-pentanediol diisobutyrate	6846-50-0	/	1871	C16H30O4	286.41		MS, RI
35	propanoic acid, 2-methyl-, 2-ethyl-1-propyl-1,3-propanediyl ester	74367-30-9	1879	1863	C16H30O4	286.41		MS, RI
36	benzoic acid, 4-ethoxy-, ethyl ester	23676-09-7	/	2173	C11H14O3	194.23		MS, RI
	**Aromatic**							
37	p-xylene	106-42-3	1138	1123	C8H10	106.17		MS, RI
38	1,3-dimethyl-benzene	108-38-3	1143	1128	C8H10	106.17	plastic	MS, RI
39	3,6,9-triethyl-3,6,9-trimethyl-tTetracyclo [6.1.0.0(2,4).0(5,7)]nonane	78578-97-9	/	1977	C18H30	246.43		MS, RI
40	o-xylene	95-47-6	1186	1173	C8H10	106.17		MS, RI
41	tetradecane	629-59-4	1400	1399	C14H30	198.39		MS, RI
42	naphthalene	91-20-3	1746	1741	C10H8	128.17	pungent	MS, RI
	**Pyrazine**							
43	2,5-dimethyl-pyrazine	123-32-0	1320	1319	C6H8N2	108.14	chocolate	MS, RI

^1^ Indicates the retention index of records in the NIST library. ^2^ Indicates the determination of retention index by headspace solid-phase microextraction gas chromatography-mass spectrometry (DB-WAX, 30 m × 250 μm, 0.25 μm film thickness). ^3^ Indicates the molecular weight of compound. ^4^ Volatile odor information comes from The Good Scents Company Information System [20].

#### 3.1.2. Quantitative Analysis of Volatiles in Sea Cucumber Samples

There is often a close relationship between the concentration of volatiles in food matrices and flavor perception. The differences in the concentration of volatiles in different parts of sea cucumber samples may reflect the varying intensity of flavors in each part. As illustrated in Figure 1d–f, the concentration of volatiles in the skin of each sea cucumber sample group was significantly lower (*p* < 0.05) compared to other parts of the samples. Specifically, the concentrations were 114.83 mg/kg for SCXs, 45.33 mg/kg for SCYs, and 424.58 mg/kg for SCZs. The concentrations of volatiles in the muscle tissues of the sea cucumber samples were as follows: SCXm (210.47 mg/kg), SCYm (73.54 mg/kg), and SCZm (797.16 mg/kg). It can be inferred that the concentration of volatile substances in muscle tissue is the highest among all parts. When the volatile contents of the skin, muscle, and tendon layers were converted into total contribution values based on their proportional mass within the whole organism, muscle tissues accounted for 60.5–74.4% of total volatiles, confirming that the edible muscle layer may serve as a major reservoir of aroma compounds in scallion-braised sea cucumber. This finding is consistent with the structural characteristics of sea cucumber tissue. The body wall is composed of a collagen-rich, highly porous matrix that not only facilitates efficient migration and absorption of exogenous volatiles from seasonings during thermal treatment but also promotes enrichment and retention of flavor compounds. Accordingly, subsequent analyses in this study focus primarily on muscle tissues as the core component governing flavor accumulation.

Alcohols were major contributors to volatile abundance in all samples, generated mainly through fatty acid oxidation (enzymatic and non-enzymatic), reduction of carbonyl compounds, and lipid degradation reactions [21]. In SCZm, SCXm, and SCYm samples, alcohol levels reached 545.99, 167.33, and 18.28 mg/kg, respectively—significantly higher than those in other tissues (*p* < 0.05). Alcohols accounted for 23.55%, 73.75%, and 65.87% of total volatiles in the three sample groups, exerting a dominant influence on the observed quantitative differences. This difference may be closely related to the intensity of thermal processing and the treatment methods of oils in the three groups of processes. Both SCX and SCZ use high-temperature oil treatment, which may promote lipid oxidation processes, potentially leading to increased formation of alcohols [22]. Among them, SCZ, due to its multi-step processing, may cause some of the alcohols inside to further participate in esterification or condensation reactions to form ester substances, which in turn makes the content of SCZ slightly lower than that of SCX [23]. The thermal processing method used in the SCY group is relatively mild to a certain extent. Therefore, the degree of lipid oxidation in this group may not be as intense as the previous two groups. The content of alcohols is 18.28 mg/kg, which is significantly lower than the other two groups.

In addition to alcohols, all samples contained esters and aromatic compounds. Esters can contribute creamy or full-bodied notes at appropriate concentrations. Meanwhile, several aromatic compounds detected in this study have previously been reported in dried SCs [17], suggesting that they may be linked to endogenous precursors. However, the relatively lower levels of esters and aromatic compounds in SCX and SCZ may indicate a reduced contribution to their overall aroma characteristics, whereas SCY exhibited a more balanced distribution of volatile classes, which may be associated with a comparatively more complex aroma profile.

A similar pattern was observed for alkenes and aldehydes. Alkenes, often associated with “grassy” or “spicy” notes [24], are unlikely to originate from sea cucumber tissues alone but are instead derived from the thermal cracking and transformation of scallions, ginger, garlic, and other Allium-based seasonings during oil heating [25]. Aldehydes, known for their low odor thresholds, are important contributors to flavor perception [26]. However, aldehydes formed during lipid oxidation can further react with amino compounds produced by protein thermal degradation through Maillard pathways, leading to the generation of furans [27], which impart nutty and roasted characteristics [28]. In the SCZ samples, furans accounted for 17.75% of total volatiles, while aldehydes represented only 2.42%, suggesting that a considerable proportion of aldehydes may have been converted to furans during heating. Overall, the scallion-braising process appears to involve multiple interacting pathways, including lipid-related transformations, Maillard-type reactions, and the incorporation of exogenous volatile compounds. The structural characteristics of sea cucumber tissues may facilitate both the retention and accumulation of these compounds, thereby contributing to the observed aroma profile. These interpretations should be considered as associative and hypothesis-generating.

#### 3.1.3. Analysis of Flavor Profile Differences Among Various Parts of Sea Cucumber Samples

Principal component analysis (PCA) is a widely used unsupervised multivariate statistical technique that reduces the dimensionality of complex datasets and facilitates visualization of intrinsic sample variation [29]. As shown in Figure 1g–i, the first two principal components of the PCA score plots for the three groups of sea cucumber samples accounted for 75.4%, 80.4%, and 87.6% of the total variance, respectively, with an average exceeding 80%. This indicates that the model exhibits strong stability and predictive power. The score plots show a tendency for samples from different tissues to occupy distinct regions, suggesting differences in their volatile profiles. The distance between sample points reflects the degree of similarity, with greater distances indicating larger compositional differences. These results suggest that, although samples originate from the same sea cucumber group, different tissues exhibit distinct flavor-related characteristics.

### 3.2. Comparison of Flavor Differences in Muscle Tissue of Sea Cucumber Samples

**Figure 2:**
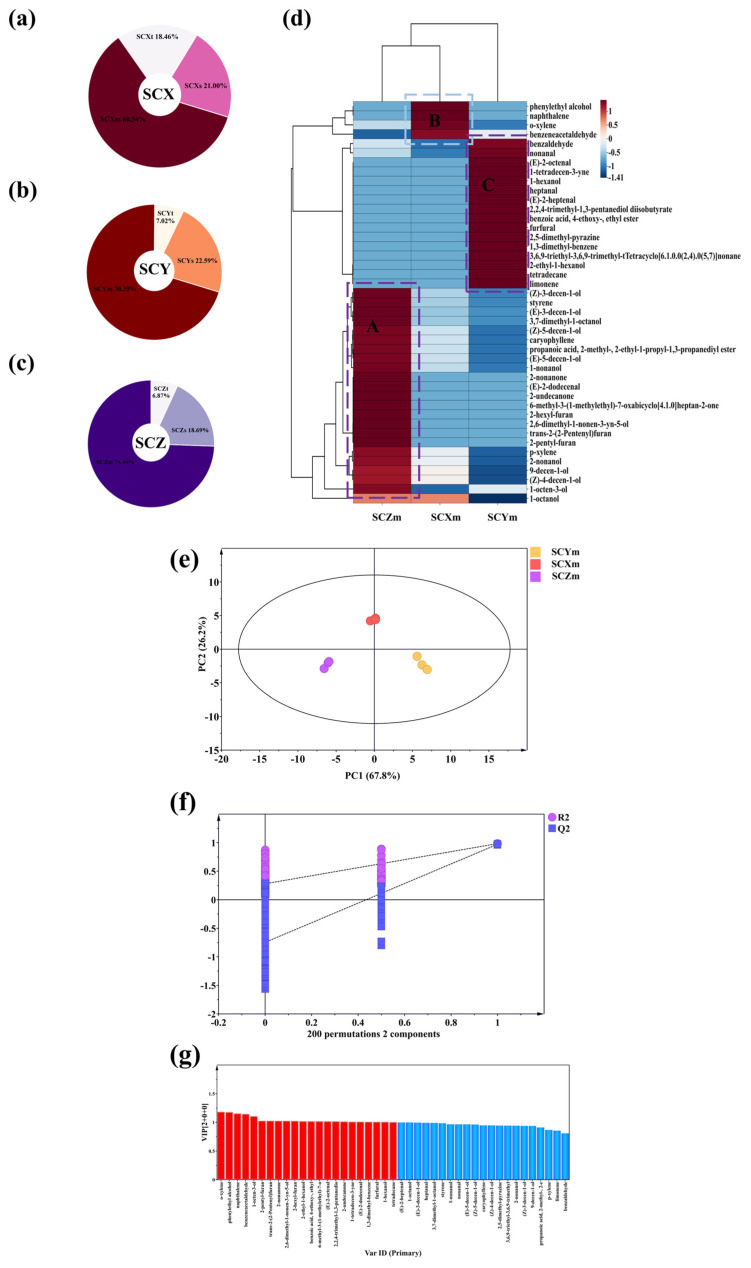
Statistical analysis of the differences in volatile compounds in different parts of scallion-braised sea cucumber. (**a**–**c**) VOCs distributions in the sea cucumber samples. (**d**) Clustering thermogram of volatile compounds in the scallion-braised sea cucumber. Boxes A–C mark three regions where volatiles showed significantly higher concentrations in one sample group (SCZm, SCXm, and SCYm,) relative to the others. (**e**) Orthogonal partial least squares analysis score plot. (**f**) Orthogonal partial least squares analysis permutation test. (**g**) Screening of key difference VOCs in sea cucumber samples.

To estimate the relative contribution of different tissues to the overall volatile profile of scallion-braised sea cucumber, the volatile concentration of each tissue was weighted according to its proportional mass within the whole organism. This calculation reflects the actual quantitative contribution of volatiles originating from each tissue. As shown in Figure 2a–c, muscle tissues exhibited the highest total volatile load in all sample groups, accounting for 70.4%, 60.5%, and 74.4% of the total volatiles in SCY, SCX, and SCZ, respectively. As the primary edible portion and a major site for volatile accumulation, the muscle layer may represent a key contributor to the overall flavor profile of scallion-braised sea cucumber. Based on this observation, subsequent comparative analyses in this study focus on muscle tissues to facilitate the characterization of inter-group differences in volatile composition.

Z-score normalization was applied to the volatile concentrations in muscle tissues to enable direct comparison among samples, and hierarchical clustering revealed three distinct distribution patterns (Regions A–C) (Figure 2d). Each region contained volatiles that showed markedly higher concentrations in a specific sample group, indicating differentiated accumulation and transformation of flavor compounds under different processing conditions. Among the three groups, SCZ contained the highest number of highly enriched volatiles (21), followed by SCY (14), while SCX exhibited relatively fewer high-abundance compounds (4). These differences may be associated with variations in raw material properties and processing conditions during braising, consistent with previous findings that processing parameters can influence flavor characteristics in aquatic products [15]. The observed differences among the three groups may also be related to the complexity of their processing procedures. The preparation process of the SCZ group involves a multi-stage compound process, starting with steaming, followed by oiling, and finally stewing. This complex process may have achieved the full release of endogenous precursors, the stepwise accumulation of lipid oxidation and the adsorption of seasonings. Thus, this group accumulated the largest number of highly enriched flavor substances. The SCY group only underwent a slow stewing process. The long-term thermal processing may have led to the effective enrichment of its flavor substances, forming a moderate number of stable compounds. In contrast, the SCX group underwent intense thermal processing for a short time. This may have led to significant evaporation of a large number of compounds, with only a few alcohols being highly enriched. Overall, these observations suggest that differences in processing conditions may be associated with distinct patterns of volatile accumulation among the three product types.

To further differentiate samples and determine the key compounds responsible for group separation, an orthogonal partial least squares-discriminant analysis (OPLS-DA) model was constructed. OPLS-DA is a supervised exploratory tool for pattern recognition and feature identification, which enhances discrimination by separating inter-group variation from intra-group noise, improving interpretability compared with PCA [30]. As shown in Figure 2e, the three sample groups tend to occupy distinct regions in the score plot, suggesting compositional differences in volatile profiles among the samples. The model exhibited high goodness-of-fit parameters (R^2^X = 0.940, R^2^Y = 0.982) and a high cumulative Q^2^ value (Q^2^ = 0.970). Model validation was further performed using permutation testing (n = 200), in which the R^2^ and Q^2^ values of permuted models were consistently lower than those of the original model, and the Q^2^ intercept was negative, supporting that the model was not a result of random overfitting [31]. In addition, CV-ANOVA indicated that the model was statistically significant (*p* = 7.05 × 10^−5^). Despite these validation results, it should be noted that the sample size in this study is limited and that the samples were derived from commercial products with multiple confounding factors. Therefore, the OPLS-DA model is interpreted here as an exploratory tool for describing group separation patterns rather than a predictive model. Accordingly, the identified discriminant metabolites should be considered as candidate features associated with sample differences rather than definitive biomarkers.

Variable Importance in Projection (VIP) scores were calculated to identify compounds contributing to group differentiation. A total of 22 volatiles showed VIP > 1, including 5 alcohols, 4 aromatics, 4 aldehydes, 3 furans, 3 ketones, 2 esters, and 1 alkene (Figure 2g). These compounds represent the main contributors to the observed variation among sample groups and provide a basis for subsequent OAV analysis and metabolite-level interpretation.

### 3.3. Identification and Analysis of the Key Flavor Actives (OAV > 1)

Odor activity value (OAV) analysis was used to estimate the potential contribution of volatile compounds based on their concentrations and reported odor thresholds. It should be noted that the odor thresholds used in this study were obtained from literature values rather than determined within the specific food matrix. Matrix effects, interactions among compounds, and differences in experimental conditions may influence the actual sensory contribution of individual volatiles. Consequently, the identified compounds with OAV > 1 are considered potential key contributors rather than definitive determinants of the overall aroma profile.

**Figure 3:**
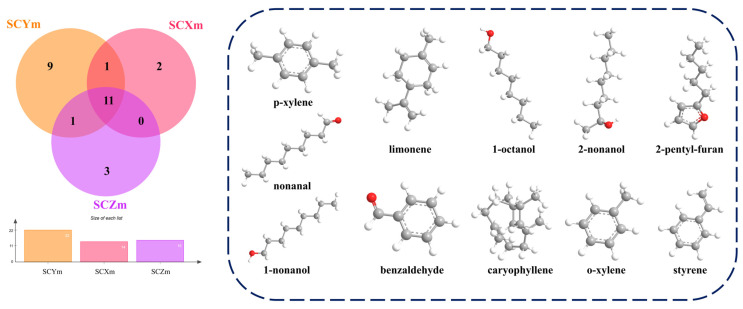
Venn diagram of key characteristic flavor compounds (OAV > 1). Different colors correspond to different sample groups, and the numbers in overlapping regions represent common compounds.

OAV analysis identified 27 key volatile compounds (OAV > 1) contributing to the flavor of scallion-braised SCs [32]. Among them, SCYm, SCXm, and SCZm contained 22, 14, and 15 active volatiles, respectively, indicating that SCY possessed a more complex aroma profile (Table 2). A total of eleven volatile compounds—including limonene, nonanal, 1-octanol, benzaldehyde, 2-pentylfuran, and caryophyllene—were detected across all sample groups, suggesting that they may represent common contributors to the characteristic aroma profile (Figure 3). These compounds are likely associated with multiple thermal and compositional processes. For example, terpenoids such as limonene are generally linked to the transformation of Allium-derived seasonings, while long-chain alcohols and aldehydes (e.g., 1-octen-3-ol and nonanal) are commonly associated with lipid oxidation processes. Aromatic compounds such as benzaldehyde are typically related to amino acid degradation and Maillard-type reactions. Terpenoids such as limonene originate mainly from thermal degradation of Allium seasonings and impart fresh spicy notes, while long-chain alcohols and aldehydes such as 1-octen-3-ol and nonanal are typical products of lipid oxidation. Aromatic compounds such as benzaldehyde derive from amino acid degradation and Maillard reactions. These results demonstrate that the flavor of scallion-braised sea cucumber is jointly shaped by lipid oxidation, protein and peptide degradation, and sugar–amino thermal reactions rather than a single biochemical pathway. However, it should be noted that the OAV analysis estimates the potential aroma contribution based on concentration and olfactory threshold, but it fails to take into account the matrix effect of the sample or the synergistic effect between compounds. Moreover, since the samples used are from commercial channels and there are various interfering variables, these explanations are only applicable to this batch of products. For the conclusion on the flavor mechanism of seasoned sea cucumbers in complex systems, further extensive research is needed to confirm it.

### 3.4. Identification of Differential Metabolites in Different SCs

#### 3.4.1. Nonvolatile Metabolite Characterization and Multivariate Statistical Analysis

**Figure 4:**
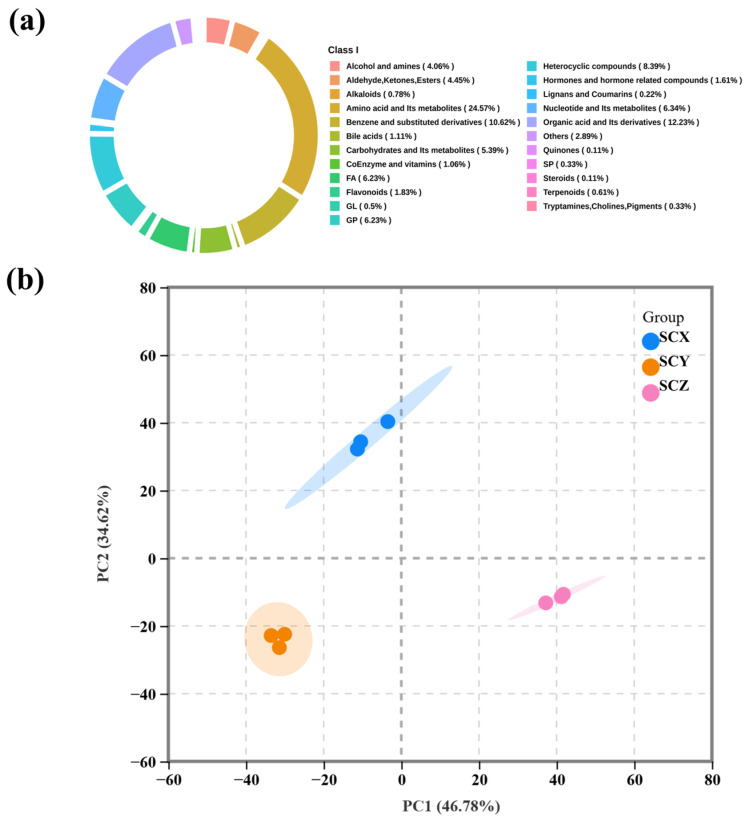
Identification of nonvolatile metabolites in scallion-braised sea cucumber. (**a**) Ring chart of the proportion of all metabolite categories. (**b**) principal component analysis score plot.

A widely targeted metabolomics approach was applied to characterize nonvolatile compounds in sea cucumber muscle tissues. A total of 1792 metabolites spanning 23 chemical categories were identified, among which amino acids and their derivatives accounted for the largest proportion (24.57%), followed by organic acids (12.23%), benzene and substituted derivatives (10.62%), heterocyclic compounds (8.39%), nucleotides (6.34%), fatty acids (6.23%), and glycerophospholipids (6.23%) (Figure 4a and Table S1). The dominance of amino acids and related intermediates reflects the highly proteinaceous composition of sea cucumber muscle and the prevalence of protein hydrolysis and amino acid degradation as major biochemical processes during thermal cooking.

Although the same 23 categories were detected across all scallion-braised sea cucumber samples, their relative abundances differed markedly, indicating compositional divergence among products. To visualize these differences, PCA was performed using all metabolites (Figure 4b). The first two principal components explained 81.40% of the total variance, demonstrating strong data representation capability. SCX, SCY, and SCZ samples clustered into distinct regions in the score plot, indicating clear metabolic separation among groups and highlighting differences arising from raw material variability, seasoning absorption, and processing conditions [33].

#### 3.4.2. OPLS-DA Analysis

**Figure 5:**
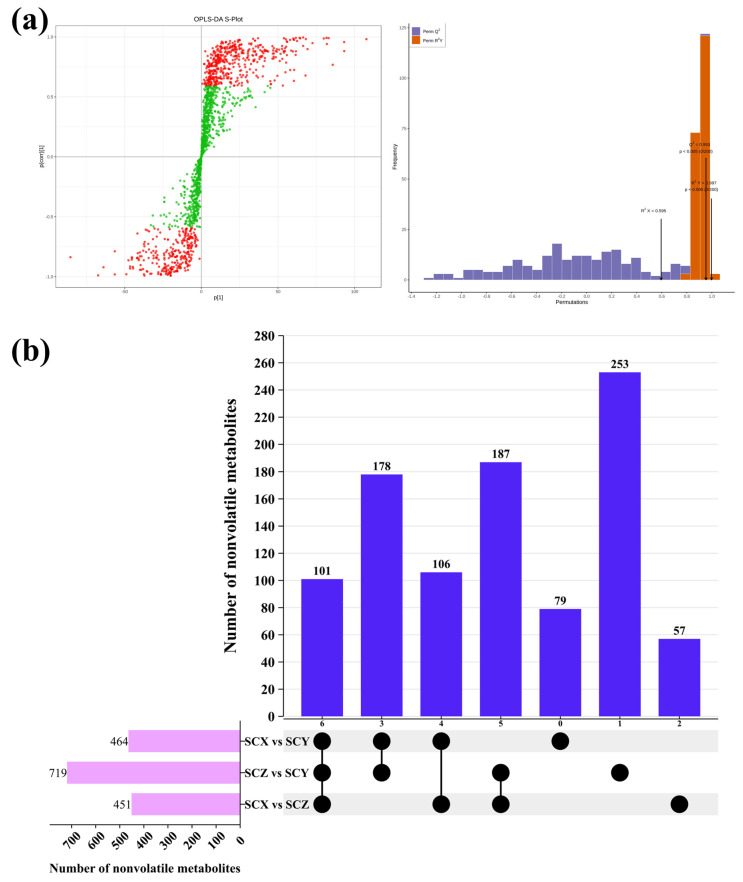

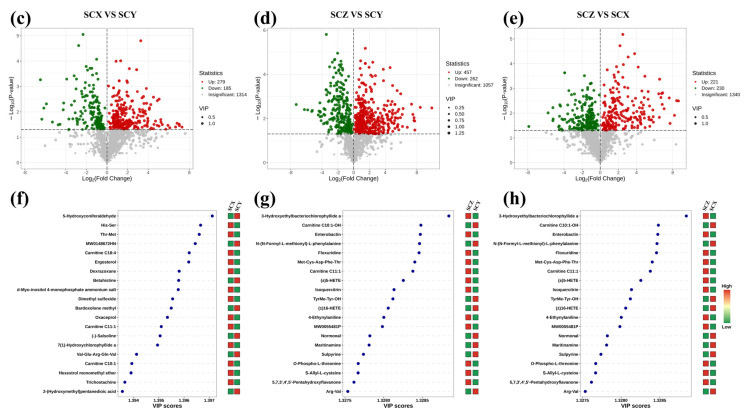
(**a**) S-plots and validation chart of the OPLS-DA model for scallion-braised sea cucumber. Red indicates potential markers with greater contribution to model discrimination, while green indicates variables with smaller contribution or non−significant variables. (**b**) Upset plot of the specific differential metabolites for three comparison groups. (**c**–**e**) Volcano plot of the differential nonvolatile metabolites of SCY vs. SCX (**c**), SCY vs. SCZ (**d**), and SCX vs. SCZ (**e**), the criteria set at VIP ≥ 1, FC ≥ 2 or ≤0.5. Red and green dots represent upregulated and downregulated differential metabolites, respectively; gray dots represent nondifferential metabolites. Top 20 compounds with highest VIP scores of SCY vs. SCX (**f**), SCY vs. SCZ (**g**), and SCX vs. SCZ (**h**).

To further dissect inter-group variation and identify key differential metabolites, an OPLS-DA model was constructed. The model exhibited excellent fitness and predictive power (R^2^X = 0.595, R^2^Y = 0.997, Q^2^ = 0.953, *p* < 0.005), and the score plot revealed complete separation among the three sample groups, confirming substantial metabolic divergence. In the corresponding S-plot (Figure 5a), metabolites located in the upper-right and lower-left regions represented the most influential contributors to sample discrimination, and compounds with VIP > 1 were classified as key differentiating variables.

Quality control (QC) samples were prepared by pooling equal aliquots from all samples and were analyzed periodically throughout the analytical sequence to monitor system stability and reproducibility. Prior to multivariate analysis, raw metabolomics data were preprocessed, including peak alignment, normalization, and filtering. Data were normalized to internal standards to correct for analytical variation. Subsequently, log transformation and unit variance (UV) scaling were applied to improve data comparability and approximate normal distribution before PCA and OPLS-DA. To further evaluate the reliability and stability of the analytical data, additional QC assessments were performed. As shown in Figure S2, QC samples were tightly clustered in the PCA score plots across all sample groups, indicating good analytical reproducibility and instrumental stability. The overall clustering pattern of all samples is presented in Figure S3, which further supports the consistency and comparability of the dataset. In addition, the coefficient of variation (CV) distribution of detected metabolites is shown in Figure S4. The majority of metabolites exhibited relatively low CV values, suggesting acceptable analytical variation and good data quality. These results collectively demonstrate the robustness and reliability of the metabolomics data used in this study.

As shown in Figure 5b, a total of 619 differential metabolites (VIP ≥ 1, FC ≥ 2 or ≤0.5, *p* ≤ 0.05) were identified across pairwise comparisons, demonstrating that scallion-braising introduces extensive biochemical variation in sea cucumber tissues [34]. These differential metabolites were mainly enriched in amino acids and their derivatives, organic acids, fatty acids, nucleotides, heterocyclic compounds, and carbohydrates—compound classes closely associated with protein degradation, lipid oxidation, nucleotide turnover, and thermal reactions. Between SCX and SCY (Figure 5c–e), 464 metabolites were significantly altered (279 upregulated and 185 downregulated), with amino acids and their derivatives representing the largest group. Major changes in aspartate, glutamate, glycine, alanine, serine, and proline—key flavor-related amino acids in sea cucumber—indicate notable differences in protein hydrolysis progression between the two products. Amino acids and their metabolites are one of the important differential metabolites between the SCX and SCY groups. Amino acids are one of the essential components of SCs, among which Aspartate, glutamate, glycine, alanine, serine, and proline are the primary amino acids that determine the flavor of SCs [35]. Between SCY and SCZ, 719 metabolites (457 upregulated and 262 downregulated) showed significant differences, with amino acids, fatty acids, and nucleotides being the dominant classes. These differences may be associated with variations in processing conditions, which have been reported to influence the transformation of unsaturated fatty acids such as EPA and DHA, as well as the generation of small-molecule metabolites [36,37]. Between SCZ and SCX, 451 metabolites (221 upregulated and 230 downregulated) were significantly different. Several distinctive biomarkers, including 3-hydroxyethylbacteriochlorophyllide, enterobactin, and glycyrrhizinate, were enriched in SCZ, which may be related to differences in the composition of scallion-derived seasonings among product sources.

Collectively, the metabolomics results suggest that scallion-braised sea cucumber undergoes complex biochemical changes involving amino acid-related processes, organic acid metabolism, and lipid-associated transformations. These differential metabolites provide a basis for subsequent integration with volatilomics and pathway-level analyses.

**Figure 6:**
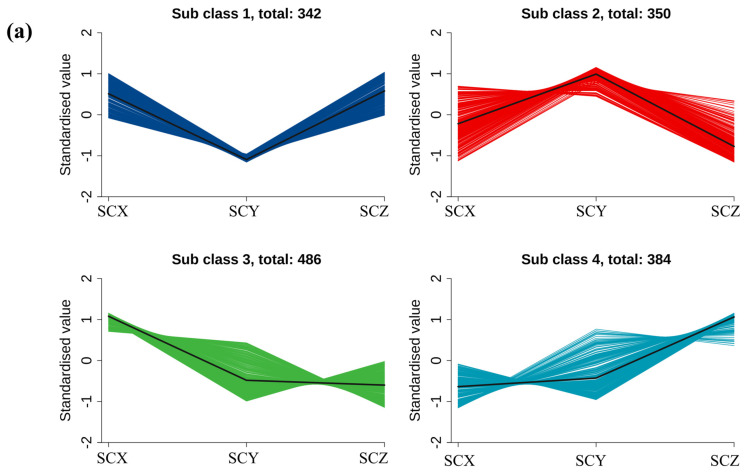

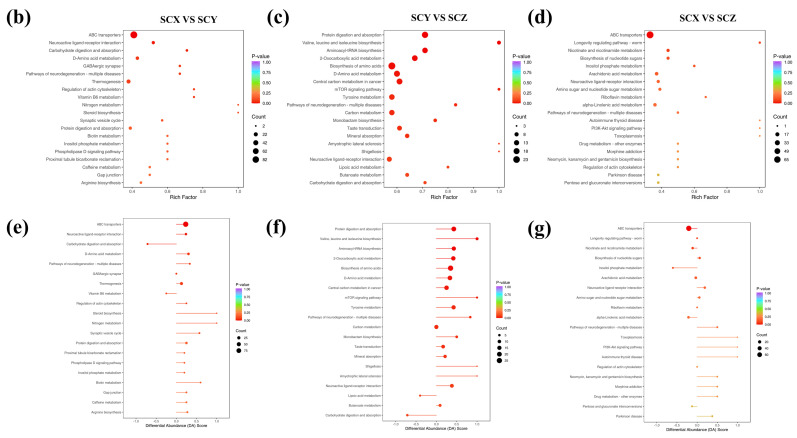
(**a**) Kmeans clustering algorithm analysis of metabolites in different scallionbraised sea cucumber. (**b**–**d**) KEGG annotations and enrichment of differentially expressed metabolites of each pairwise comparison of scallionbraised sea cucumber. (**e**–**g**) differential abundance (DA) score of differentially expressed metabolites of each pairwise comparison of scallionbraised sea cucumber.

K-means clustering further clarified the metabolic distinctions among the three products by grouping 619 differential metabolites into four subclasses with clear distribution patterns (Figure 6a and Table S2). Subclasses 1 and 3, which were significantly enriched in SCY and dominated by amino acids, carbohydrates, and organic acids, suggest more extensive protein hydrolysis and sugar turnover, providing abundant precursors for Maillard and Strecker reactions. In contrast, subclasses 2 and 4 showed higher levels of amino acids, fatty acids, glycerophospholipids, and benzene derivatives in SCX and SCZ, indicating stronger lipid oxidation and membrane degradation during processing. Across all subclasses, amino acids and related intermediates represented the largest metabolite category, consistent with the protein-rich composition of sea cucumber muscle. When considered together with volatilomic and correlation analyses, these findings suggest that the observed flavor differences among products may be associated with variations in metabolic pathways, with SCY showing relatively greater involvement of protein and carbohydrate-related processes, and SCX and SCZ showing relatively stronger lipid-associated transformations [38].

### 3.5. KEGG Annotation and Enrichment Analysis of Differential Metabolites

KEGG pathway enrichment analysis was conducted to elucidate the biochemical processes underlying differential metabolite variation among scallion-braised sea cucumber samples. The top 20 enriched pathways are shown in Figure 6b–g. Among them, amino acid biosynthesis and protein digestion and absorption were the most significantly enriched pathways, reflecting the predominance of protein hydrolysis and amino acid turnover during heating. This agrees with the metabolite composition data, in which amino acids and their derivatives were the largest metabolite class.

Carbohydrate-related pathways—including galactose metabolism, fructose and mannose metabolism, and 2-oxocarboxylic acid metabolism—were also significantly enriched, indicating that sugar degradation and associated energy metabolism contribute to the generation of Maillard and Strecker reaction precursors under thermal processing. Additionally, lipid-related pathways showed marked enrichment, consistent with the observed accumulation of fatty acids, glycerophospholipids, and downstream lipid oxidation products. The ABC transporter pathway was enriched in several comparisons (e.g., SCY vs. SCX and SCX vs. SCZ). However, this pathway is interpreted here as a database-derived classification rather than a direct functional mechanism in the context of food processing. Its enrichment likely reflects the inclusion of metabolites categorized under transport-related pathways in KEGG, rather than indicating a direct role in aroma formation.

Differential abundance (DA) score analysis was used to further characterize pathway-level variation by evaluating the overall direction and magnitude of metabolite changes. Pathways with relatively high DA scores—such as steroid biosynthesis, nitrogen metabolism, and carbohydrate digestion and absorption—showed notable variation among commercial products, suggesting differences in the representation of protein-, lipid-, and carbohydrate-related metabolites.

Overall, the combined results from enrichment analysis, *p*-values, and DA scores suggest that differential metabolites are primarily distributed across pathways related to amino acids, carbohydrates, and lipids. Based on the association explanations within the dataset, the enrichment analysis and differential analysis scores indicate that the differentially expressed metabolites are mainly distributed in the metabolic pathways related to amino acids, carbohydrates, and lipids.

### 3.6. Correlation Between Volatiles and Nonvolatile Metabolites

**Figure 7:**
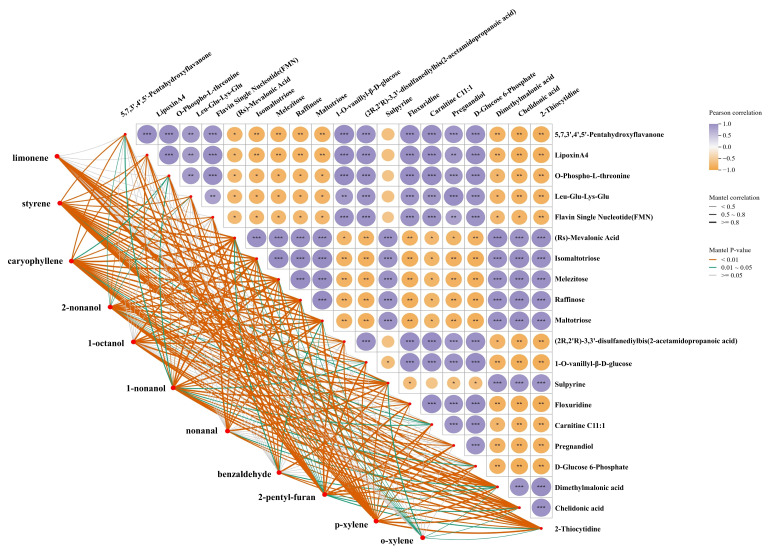
Correlation analysis between key volatiles (OAV > 1) and differential metabolites (Top 20 with the highest VIP scores) scallion-braised sea cucumber. * Indicates the significance level of the Mantel test correlation: * *p* < 0.05, ** *p* < 0.01, *** *p* < 0.001.

To investigate the biochemical basis of key aroma formation in scallion-braised sea cucumber, Spearman correlation analysis was performed between aroma-active volatiles (OAV > 1) and differential nonvolatile metabolites identified through widely targeted metabolomics. A total of 619 differential metabolites were initially screened. To further prioritize the most influential variables, metabolites were ranked based on their variable importance in projection (VIP) scores derived from the OPLS-DA model, and the top 20 metabolites were selected for subsequent correlation analysis. The resulting correlation network (Figure 7) revealed clear metabolic sources and transformation pathways contributing to the generation of characteristic flavor compounds under the multi-component cooking system.

Amino acids, peptides, and protein degradation intermediates showed strong positive correlations (|r| > 0.70, *p* < 0.05) with multiple aldehydes, alcohols, and heterocyclic compounds such as nonanal, 2-pentylfuran, dimethyl sulfide, and styrene. These relationships suggest that thermal degradation of collagen-rich structural proteins in the sea cucumber body wall produced free amino acids and short peptides, which subsequently underwent Strecker degradation, Maillard reactions, and interaction with oil- and scallion-derived compounds to form characteristic balsamic, umami, and roasted notes [39]. This supports the view that protein digestion and amino acid metabolism are major driving pathways of volatile formation during braising, consistent with mechanisms widely observed in heat-processed aquatic and meat products [40,41].

Fatty acid oxidation products, including linoleic acid and oleic acid derivatives, exhibited significant positive correlations with 2-pentylfuran, nonanal, heptanal, and other lipid-derived volatiles (|r| > 0.70). 2-Pentylfuran and aldehydes are typical markers of linoleic acid oxidation [42]. These associations indicate that thermal oxidation of endogenous membrane lipids, as well as interaction with cooking oil, contributed substantially to the production of fatty, nutty, roasted, and waxy aroma notes.

Carbohydrate-related metabolites, including glucose and intermediates of glycolysis and the pentose phosphate pathway, were also correlated with heterocyclic volatile compounds. These relationships suggest that sugar-related metabolites may be associated with the formation of thermally derived aroma compounds, consistent with the known involvement of Maillard reactions and caramelization in cooked foods [43].

Integrating volatilomics, nonvolatile metabolite profiling, and metabolic pathway correlations, this study demonstrates that the characteristic aroma of scallion-braised sea cucumber is generated through the coordinated transformation of proteins, lipids, and carbohydrates during high-temperature cooking under a multi-seasoning system. Protein degradation supplies amino acids and peptides that participate in Strecker and Maillard reactions to form aroma-active compounds; lipid oxidation contributes aldehydes and furans responsible for roasted and waxy notes; and carbohydrate degradation combined with thermal reactions enhances heterocyclic aroma formation. These findings reveal, for the first time, that flavor development in real braising conditions is not governed by a single precursor pathway but by multi-dimensional metabolic coupling, emphasizing the critical role of interactions among seasonings, endogenous substrates, and heat in shaping the final flavor characteristics of sea cucumber.

Despite the comprehensive analytical approach employed in this study, several limitations should be acknowledged. Although the combination of flavoromics and widely targeted metabolomics enables the simultaneous characterization of both volatile and non-volatile compounds, due to the observational nature of the research design and the inherent variability of the commercial samples used, the associations between metabolites are limited to the samples of this particular experiment. Future studies should focus on controlled model systems to isolate specific processing variables, incorporate sensory evaluation techniques to validate aroma contributions, and better elucidate the transformation pathways underlying flavor formation. Such approaches would further strengthen the mechanistic understanding of flavor development in complex thermally processed foods.

## 4. Conclusions

In this study, an integrated analytical approach combining flavoromics and widely targeted metabolomics was applied to systematically characterize the volatile and nonvolatile profiles of scallion-braised sea cucumber from different commercial sources. The results showed that volatile compounds were unevenly distributed among different tissues, with muscle tissues contributing the majority of total volatile content and representing the primary carrier of aroma-related compounds. Differences in volatile composition among sample groups were reflected in the relative abundance of alcohols, aldehydes, esters, and furans, suggesting variations in underlying chemical transformations associated with thermal processing and seasoning interactions. Multivariate analyses revealed distinguishable compositional patterns among sample groups. These differences were further supported by clustering analysis and metabolite distribution trends, indicating systematic variation in both volatile and nonvolatile components. A total of 43 volatile compounds and 1792 nonvolatile metabolites were identified, spanning eight and 23 chemical categories, respectively, with amino acids and their derivatives being the most abundant class. Multivariate analysis and screening criteria (VIP ≥ 1, OAV > 1, FC ≥ 2 or ≤0.5, and *p* ≤ 0.05). In parallel, KEGG pathway enrichment and DA score analyses highlighted that differential metabolites were mainly associated with amino acid-, carbohydrate-, and lipid-related pathways. Integration of volatilomic, metabolomic, and pathway correlation analyses demonstrated that the formation of characteristic flavors in scallion-braised sea cucumber is driven by coordinated biochemical processes, including protein hydrolysis, lipid oxidation, and carbohydrate degradation, rather than by a single precursor pathway. Overall, the study provides a comprehensive compositional framework for understanding flavor-related differences in scallion-braised sea cucumber and highlights the importance of integrating volatile and nonvolatile analyses in complex food matrices. These findings contribute to a better understanding of flavor variation in commercially processed sea cucumber products and may provide a basis for future studies aimed at improving product quality and standardization.

## Figures and Tables

**Table 2:** The OAV of volatiles in sea cucumber samples.

No. ^1^	Name	CAS	Odor Threshold (μg/g) ^2^	OAV		
				SCX	SCY	SCZ
	Alkenes					
1	limonene	138-86-3	0.2	17.99641	36.03712	17.78004
2	styrene	100-42-5	0.065	87.69301	26.5428	345.3985
4	caryophyllene	87-44-5	0.064	70.24429	28.95589	156.7997
	Alcohols					
5	1-hexanol	111-27-3	0.0056	0	105.8172	0
6	1-octen-3-ol	3391-86-4	0.0015	0	862.5931	2203.88
7	2-ethyl-1-hexanol	104-76-7	0.27	0	8.490455	0
9	2-nonanol	628-99-9	0.058	487.3699	80.28997	1004.067
10	1-octanol	111-87-5	0.1258	347.2628	20.51664	352.1025
11	1-nonanol	143-08-8	0.0455	220.7686	36.25372	700.5856
19	phenylethyl alcohol	60-12-8	0.56423	6.470308	0	0
	Aldehydes					
20	heptanal	111-71-7	0.00025	0	5258.329	0
21	(E)-2-heptenal	18829-55-5	0.051	0	23.96384	0
22	nonanal	124-19-6	0.0011	2628.707	5708.614	3421.781
23	(E)-2-octenal	2548-87-0	0.00034	0	3543.579	0
24	furfural	98-01-1	0.77	0	2.847515	0
25	benzaldehyde	100-52-7	0.75089	4.147645	5.995126	4.766823
26	benzeneacetaldehyde	122-78-1	0.0063	816.0793	332.2776	0
27	(E)-2-dodecenal	20407-84-5	0.0014	0	0	4755.712
	Furan					
28	2-pentyl-furan	3777-69-3	0.0058	813.1983	975.4345	21,705.65
	Ketone					
31	2-nonanone	821-55-6	0.041	0	0	61.81178
32	2-undecanone	112-12-9	0.0055	0	0	1232.22
	Aromatic					
37	p-xylene	106-42-3	1	3.327399	2.374543	4.635333
38	1,3-dimethyl-benzene	108-38-3	1	0	2.923778	0
40	o-xylene	95-47-6	0.45023	12.80851	3.760365	6.158016
41	tetradecane	629-59-4	1	0	1.662306	0
42	naphthalene	91-20-3	0.006	428.6103	0	0
	Pyrazine					
43	2,5-dimethyl-pyrazine	123-32-0	0.08	0	24.0824	0

^1^ Numbers correspond to those in records in Table 1. ^2^ Odor thresholds were referenced from a book named Odor thresholds compilations of odor threshold values in air, water, and other media (second enlarged and revised edition).

## Data Availability

The original contributions presented in this study are included in the article/Supplementary Materials. Further inquiries can be directed to the corresponding author.

## Supplementary Materials

The following supporting information can be downloaded at: https://www.mdpi.com/article/10.3390/foods15081452/s1, Table S1. List of Classification table of 1792 non-volatile metabolites. Table S2. Classification table of 619 significantly differentially non-volatile metabolites. Figure S1. Sample diagram of the scallion-braised sea cucumber. (s) The skin layer, (m) the muscle layer, (t) the tendon layer. Figure S2. PCA score plots of all samples including quality control (QC) samples. Note: PC1 represents the first principal component, PC2 represents the second principal component, and the percentages indicate the contribution rate of each principal component to the dataset; each point in the figure represents a sample. Samples within the same group are represented by the same color, and “Group” indicates the grouping. Figure S3. Overall clustering diagram of samples. Note: The horizontal axis represents sample names, the vertical axis represents metabolite information, “Group” represents groups, and different colors represent different values obtained through normalization of relative content (red indicates high content, green indicates low content). Among them, “Class” represents the first-level classification of substances; “cluster”: clustering analysis is conducted for both metabolites and samples. Figure S4. CV distribution graphs of each group of samples. The horizontal axis represents the CV value, and the vertical axis indicates the proportion of substances with CV values less than the corresponding value to the total number of substances. Different colors represent different sample groups.
