# Supplementary material for: Characterizing the Flavor Profile and Metabolite Discrepancies of Scallion Braised Sea Cucumber Body Wall by Flavoromics and Widely Targeted Metabolomics
Source: Foods. 2026 Apr 21;15(8):1452. doi: 10.3390/foods15081452 (PMC13115282; doi:10.3390/foods15081452)
Supplement: Supplementary file 1 [file foods-15-01452-s001.zip › Supporting Information.pdf]

Supporting Information

Characterizing the Flavor Profile and Metabolite Discrepancies of Scallion Braised Sea Cucumber Body Wall by Flavoromics and Widely Targeted Metabolomics

Xinran Li [1,2,†], Jiahui Song [1,†], Enhui Ma [1], Qiang Geng [1] and Songyi Lin [1,2,*]

[1] State Key Laboratory of Marine Food Processing & Safety Control, National Engineering Research Center of Seafood, School of Food Science and Technology, Dalian Polytechnic University, Dalian 116034, China; lixr101@163.com (X.L.); s1924225665@163.com (J.S.); 18741144397@163.com (E.M.); geng17352079760@163.com (Q.G.)

[2] Engineering Research Center of Special Dietary Food, The Education Department of Liaoning Province, Dalian 116034, China

* Correspondence: linsongyi730@163.com

† These authors contributed equally to this work and should be considered co-first authors.

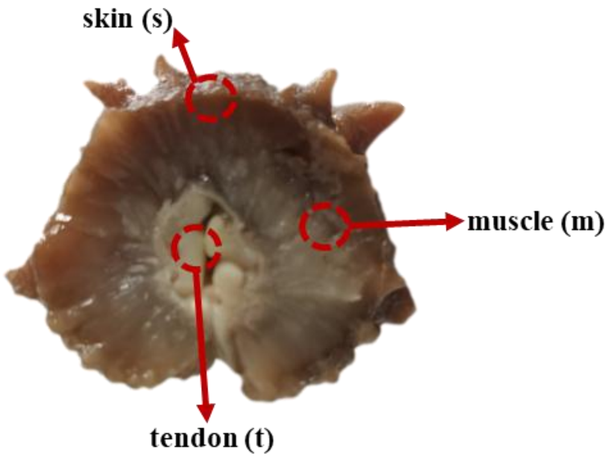

Figure S1. Sample diagram of the scallion-braised sea cucumber. (s) The skin layer, (m) the muscle layer, (t) the tendon layer.

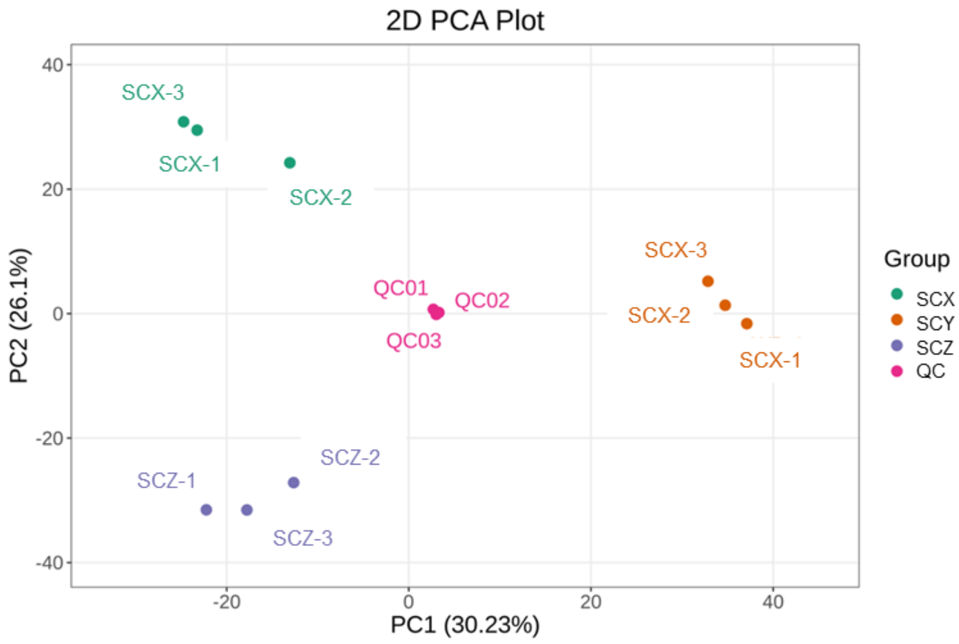

Figure S2. PCA score plots of all samples including quality control (QC) samples. Note: PC1 represents the first principal component, PC2 represents the second principal component, and the percentages indicate the contribution rate of each principal component to the dataset; each point in the figure represents a sample. Samples within the same group are represented by the same color, and "Group" indicates the grouping.

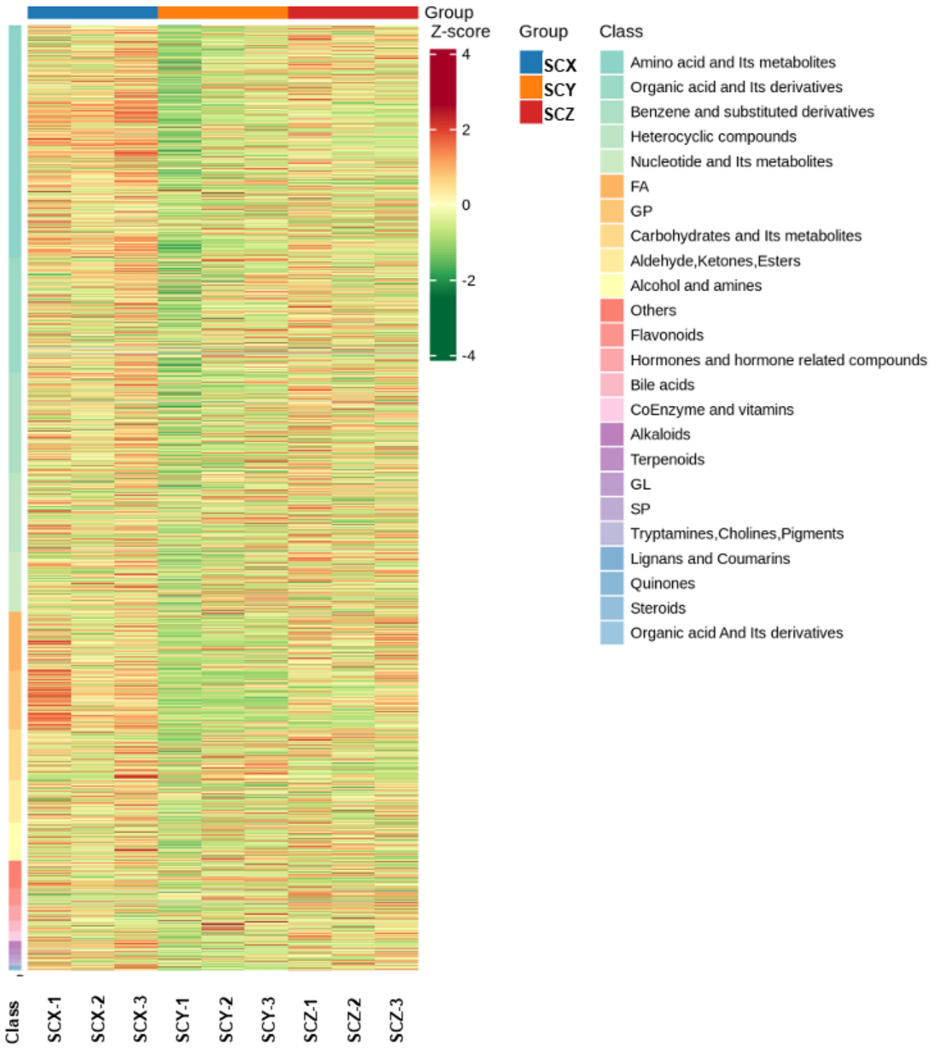

Figure S3. Overall clustering diagram of samples

Note: The horizontal axis represents sample names, the vertical axis represents metabolite information, "Group" represents groups, and different colors represent different values obtained through normalization of relative content (red indicates high content, green indicates low content). Among them, "Class" represents the first-level classification of substances; "cluster": clustering analysis is conducted for both metabolites and samples.

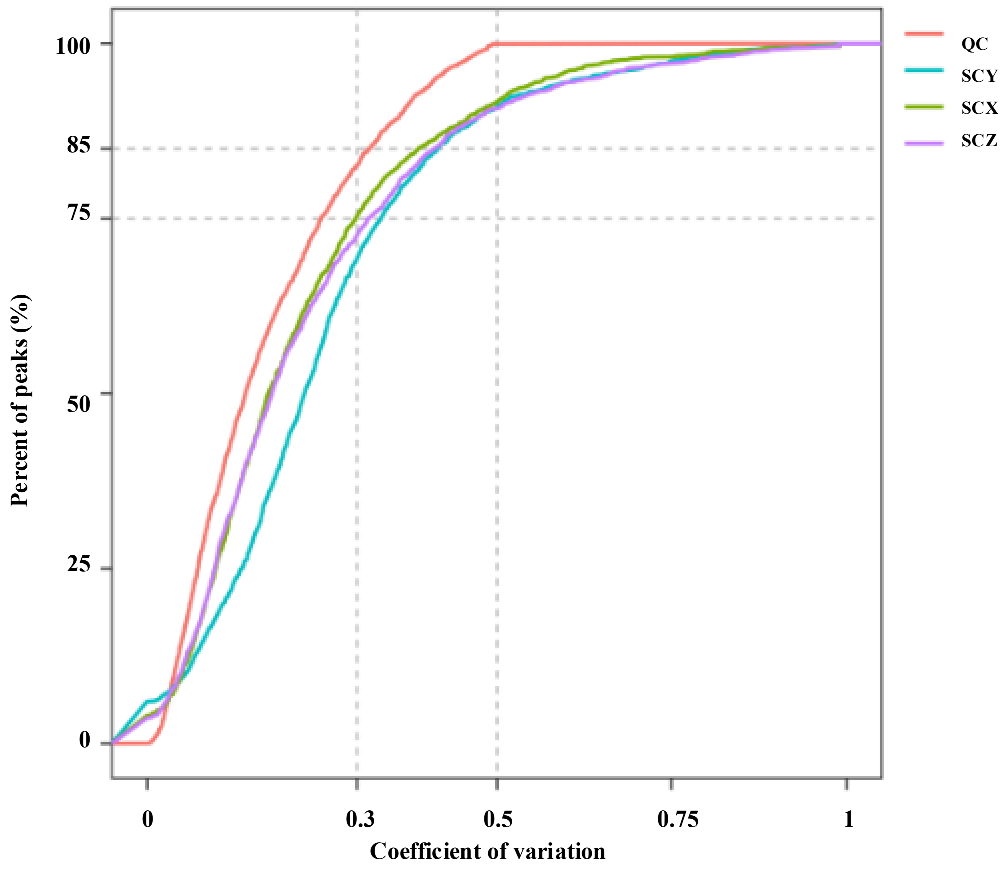

Figure S4. CV distribution graphs of each group of samples. The horizontal axis represents the CV value, and the vertical axis indicates the proportion of substances with CV values less than the corresponding value to the total number of substances. Different colors represent different sample groups.
